# Basal Plane Graphene Coated Zn Powder for High Performance Aqueous Zn Metal Batteries

**DOI:** 10.1002/advs.77287

**Published:** 2026-08-20

**Authors:** Dongju Kim, Daebeom Jeong, Kijung Kim, Van‐Chuong Ho, Jongsoon Kim, Hun‐Gi Jung, Jung Ho Kim, Goojin Jeong, Junyoung Mun

**Affiliations:** ^1^ School of Advanced Materials Science and Engineering Sungkyunkwan University Suwon‐si Gyeonggi‐do Republic of Korea; ^2^ Department of Future Energy Engineering Sungkyunkwan University Suwon‐si Gyeonggi‐do Republic of Korea; ^3^ Department of Energy Science Sungkyunkwan University Suwon Republic of Korea; ^4^ KIST‐SKKU Carbon‐Neutral Research Center Sungkyunkwan University Suwon Republic of Korea; ^5^ Energy Storage Research Center Korea Institute of Science and Technology Seoul Republic of Korea; ^6^ Institute for Superconducting and Electronic Materials (ISEM) Australian Institute for Innovative Materials University of Wollongong North Wollongong NSW Australia; ^7^ Advanced Batteries Research Center Korea Electronics Technology Institute Seongnam Republic of Korea; ^8^ SKKU Institute of Energy Science and Technology (SIEST) Sungkyunkwan University Suwon‐si Gyeonggi‐do Republic of Korea

**Keywords:** dendrite, epitaxial growth, graphene coating, metal powder electrode, solvation control

## Abstract

Aqueous zinc metal batteries (AZMBs) suffer from Zn surface degradation caused by dendrite growth, corrosion, and electrolyte side reactions. Zn powder is an essential component for flexible electrodes, enhanced electrolyte accessibility, and precise control of areal capacity. However, its application is severely limited by pronounced surface failure. Here, a graphene coating is applied to Zn powder via a shear‐stress‐driven dry process, forming a basal plane‐dominated protective layer. This coating suppresses the surface degradation by simultaneously passivating the surface, regulating the interfacial Zn^2+^ solvation structure, and establishing epitaxial Zn metal deposition toward the (002) crystallographic orientation. The resulting graphene‐coated Zn powder (Gr_Zn) exhibits stable cycling with a low voltage hysteresis of 16.1 mV over 640 h at 1 mA cm^−2^, outperforming zinc foil and bare Zn powder electrodes. Moreover, Gr_Zn delivers high‐rate capability and exceptional high areal capacity (>20 mAh cm^−2^) in full‐cell and pouch‐cell configuration.

## Graphical abstract

A mechanofusion‐driven dry coating forms a hydrophobic graphene shell on Zn powder, stabilizing the electrode‐electrolyte interface. This basal‐plane‐rich protective layer modifies the Zn^2+^ solvation environment and promotes preferential growth along the (002) plane, suppressing dendrite and parasitic reactions. As a result, the Zn anode achieves improved cycling stability and high areal capacity in aqueous batteries.

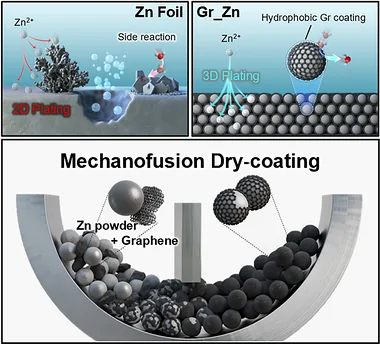

## 1 Introduction

Aqueous Zn metal batteries (AZMBs) are emerging as promising next‐generation energy storage systems to address the serious safety issues of conventional Li‐ion batteries [1, 2, 3, 4, 5, 6]. Besides, Zn is highly affordable in the Earth's crust, and Zn^2+^ is a multivalent ion providing a high theoretical capacity (820 mAh g^−1^) [7]. As the negative electrode, Zn exhibits an efficient low electrochemical equilibrium potential of −0.76 V (vs. SHE) lying within the narrow electrochemical window of aqueous electrolyte [7]. Thus, Zn metal is an inevitable candidate for constructing aqueous batteries with high‐energy density at low cost [8, 9, 10, 11]. Despite these unique advantages, the performance of AZMBs are not acceptable for practical applications owing to surface failures of Zn metal electrodes. In detail, the electrochemical deposition of Zn metal causes dendritic growth, hydroxide‐mediated corrosion, and hydrogen evolution reactions (HER) from aqueous electrolyte decomposition (Figure 1A).

**FIGURE 1:**
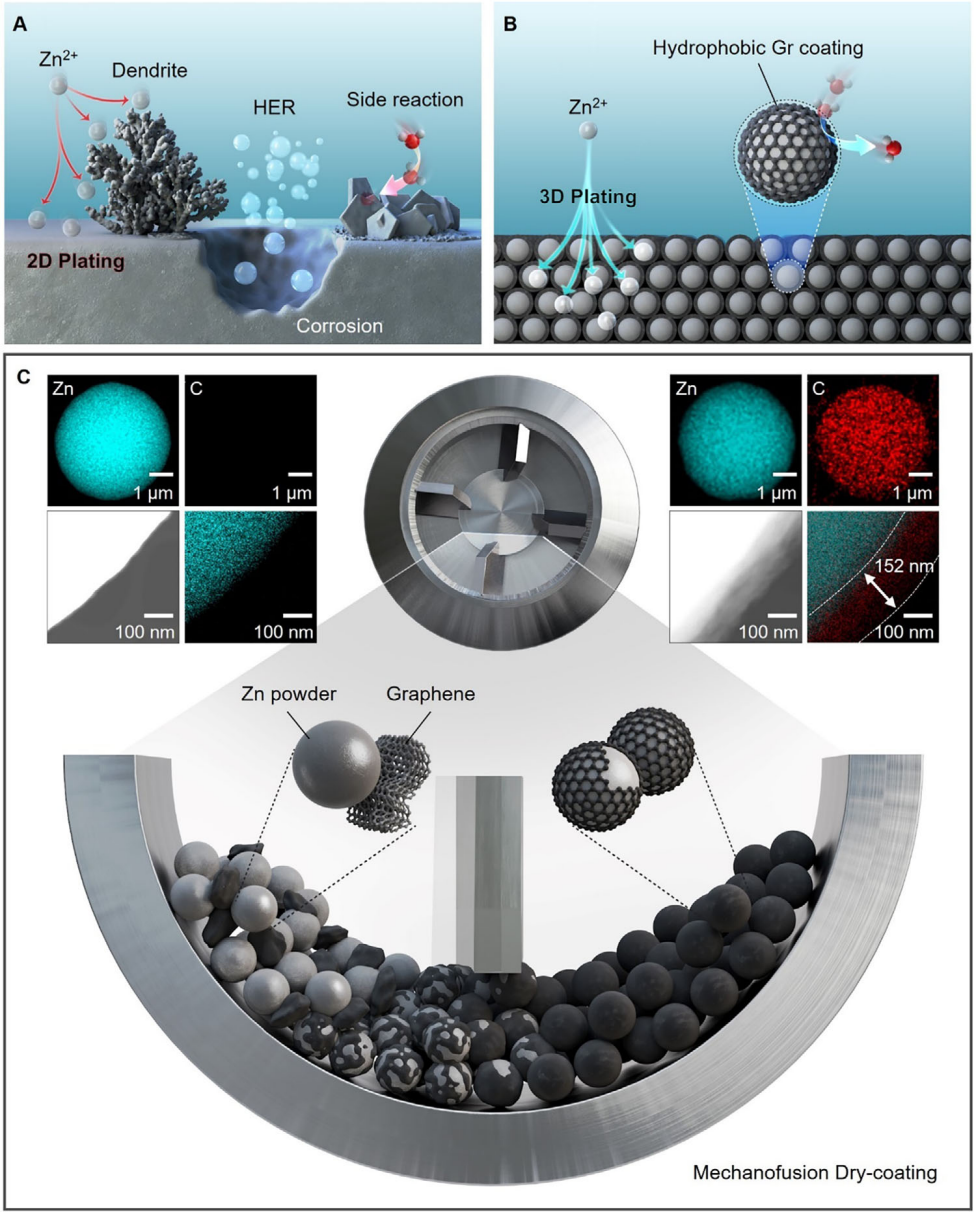
(A and B) Schematic illustrations of (A) the zinc foil and (B) the Gr_Zn electrode. (C) Mechanofusion‐based dry coating process with TEM‐EDX images of whole pristine Zn powder and its surface (upper left), and the same of the graphene‐coated Zn powder (upper right).

In order to mitigate Zn metal surface deterioration, foil‐type Zn electrodes are widely employed in AZMBs [12, 13]. The structure of foil minimizes the contact area to the electrolyte, thereby suppressing inherent Zn metal surface failures. However, this advantage simultaneously has negative effects on the feasibility of electrochemical reaction for AZMB by limiting the electrochemical active surface. Under a high current condition for AZMB, it impedes the accessibility of a large quantity of Zn ions. When thick and high areal capacity electrodes are required for achieving high energy density cells, this limitation becomes significant because the surface area of Zn foil does not increase proportionally with its thickness. Zn foil has another issue, which is a physical limitation for cell manufacturing. Zn metal exhibits a high stiffness, with a high Young's modulus of 86 GPa, which is far greater than other metal electrodes used in high‐energy‐density batteries (eg. Li = 4.9 GPa, and Na = 9 GPa) [14, 15]. Because the electrodes are installed with various form factors like pouch, prismatic, and cylinder cells, its mechanical flexibility is essential. The high stiffness of Zn foil metal restricts the adaptability to diverse cell geometries. Furthermore, coupling of the cathode and anode requires accurate electrode thickness control to deliver efficient capacity balance and minimize inactive space of cell. The conventional Zn foil fabrication usually relies on unsophisticated rolling processes, limiting fine tunability in electrode thickness. These electrochemical and engineering constraints of Zn foil electrodes provoke major challenges to the large‐scale utilizations of AZMB with high energy density and power capability [16, 17].

Zn powder (P_Zn) composite electrodes are much closer to practical cell‐manufacturing environment than Zn foil owing to their high surface area and physical flexibility. The composite electrodes have also fine‐tunability of areal capacity, which is essential for practical industry production [18, 19, 20, 21]. Additionally, the composite electrodes present wide contact areas between P_Zn and electrolyte, enabling efficient transport [22, 23]. However, P_Zn undergo far more surface side reactions than zinc foil because of its substantially larger electrolyte contact area [24, 25, 26]. The insulating byproducts generated from electrolyte side reactions deposit on the P_Zn surface, resulting in unfavorable potential polarization of AZMB. Moreover, the cell condition is unstable by changing electrolyte pH values by hydrogen gas evolution causing high cell internal pressure [27, 28]. Surface engineering of P_Zn is effective in solving these issues. By forming a robust coating on the P_Zn, the Zn‐electrolyte interface can be effectively stabilized [17, 29, 30, 31, 32].

Various surface‐coating strategies on Zn powder have been extensively investigated to mitigate its intrinsic instability in AZMBs. Conventional coatings, including inorganic oxides, polymeric layers, and carbon‐based materials, have been widely applied to suppress dendrite growth, alleviate corrosion, and reduce parasitic side reactions [33, 34]. While these approaches provide partial stabilization of the Zn surface, many coating methods rely on wet chemical and heat processes, which can cause unexpected interfacial degradations. The coating substance and conformability are restricted by the coating process influenced by surface instability of Zn powder. Consequently, developing a coating strategy that simultaneously preserves the advantages of Zn powder electrodes while ensuring robust interfacial regulation remains a critical challenge.

Herein, graphene‐coated P_Zn is developed by mechanofusion dry‐coating of graphene powder. This coating method enables the surface of P_Zn is homogeneously covered by a small amount of graphene coating material (<5 wt.%) without any additional cost‐consuming steps of solvents or post‐heat treatment. The graphene has hydrophobicity, which suppresses side reactions, together with high intrinsic electronic and thermal conductivity [35]. The functional hydrophobic coating can effectively suppress side reactions of water decomposition (Figure 1B) [36, 37, 38]. The free water molecules, which are known as electrochemically unstable is repelled from the hydrophobic surface of electrode [39, 40, 41, 42]. This approach realized long cyclability in symmetric cell (>640 h) while maintaining low voltage hysteresis (16.1 mV) compared to Zn foil as well as P_Zn. Furthermore, with the suppression of side reactions, Gr_Zn enables the simultaneous transport of a large amount of zinc ions, achieving outstanding rate capability. A millimeter‐scale, high‐loading electrode delivering over 22.7 mAh cm^−2^ was employed, in contrast to the Zn foil electrode, which can provide only about 7.5 mAh cm^−2^. This work proposed a tunable Zn anode with high‐energy density and a long lifespan, capable of fulfilling commercialization demands across various fields, including folding and bending (Figure S1), highlighting its potential for flexible and foldable device applications.

## 2 Results and Discussion

In order to coat the surface of Zn powder with graphene, a shear‐based dry coating process was employed to mixtures of Zn and graphene powder (Figure 1C). During the coating process, rotating blades impose shear stress and compressive force on particles between the inner wall and the blade path, driving graphene flakes to conformally wrap the surface of the Zn particles [43, 44, 45]. As shown in Figure 2, transmission electron microscope (TEM) and energy dispersive spectroscopy (EDS) images of pristine Zn (P_Zn), 1, 3, and 5 wt.% Gr_Zn confirm that the coating processes are feasible by presenting coating layer of P_Zn surface. High‐resolution TEM and EDS images also indicate that thin and homogeneous coating layer on the P_Zn consists of carbon layer with 83, 152, and 156 nm thickness, respectively (Figures S2 and S3). To verify that the carbon substances wrapping the Gr_Zn, the deconvoluted C1s spectra from x‐ray photoelectron spectroscopy (XPS) were analyzed (Figure S4). Unlike the carbon in P_Zn forms C = C bonding as indicated by the 285 eV peak, the carbon in all of Gr_Zn exhibits sharp peaks of sp^2^ bonding at 284.6 eV, which implies the presence of graphene on the surface of Gr_Zn [46, 47, 48]. Simultaneously, the intensity of Zn 2p spectra decreases as the graphene content increases, which represents that the graphene conformally covers the Zn particle, not island type. The surface atomic ratios of zinc to carbon of 1, 3, and 5 wt.% Gr_Zn decreases as 1.6, 0.5, and 0.5 at.%, respectively. As the graphene coating content increases, zinc becomes increasingly masked, however, at coating amounts of 3 wt.% or higher it is largely retained around 0.5 at.%, indicating the graphene content of 3 wt.% is sufficient to cover the surface of Zn powder. Scanning electron microscopy (SEM) images in Figure S5 further confirmed the coverage and uniformity of graphene on the surface of zinc. P_Zn has a rough surface, and as the graphene coating content increases up to 3 wt.%, the surface appears increasingly covered with a smoother texture. However, when the graphene coating content exceeds 3 wt.% and reaches 5 wt.%, surface agglomeration occurs, indicating that 3 wt.% Gr_Zn has the most uniform coating layer with complete coverage. Therefore, 3 wt.% was established as the optimal coating content and applied in subsequent experiments.

**FIGURE 2:**
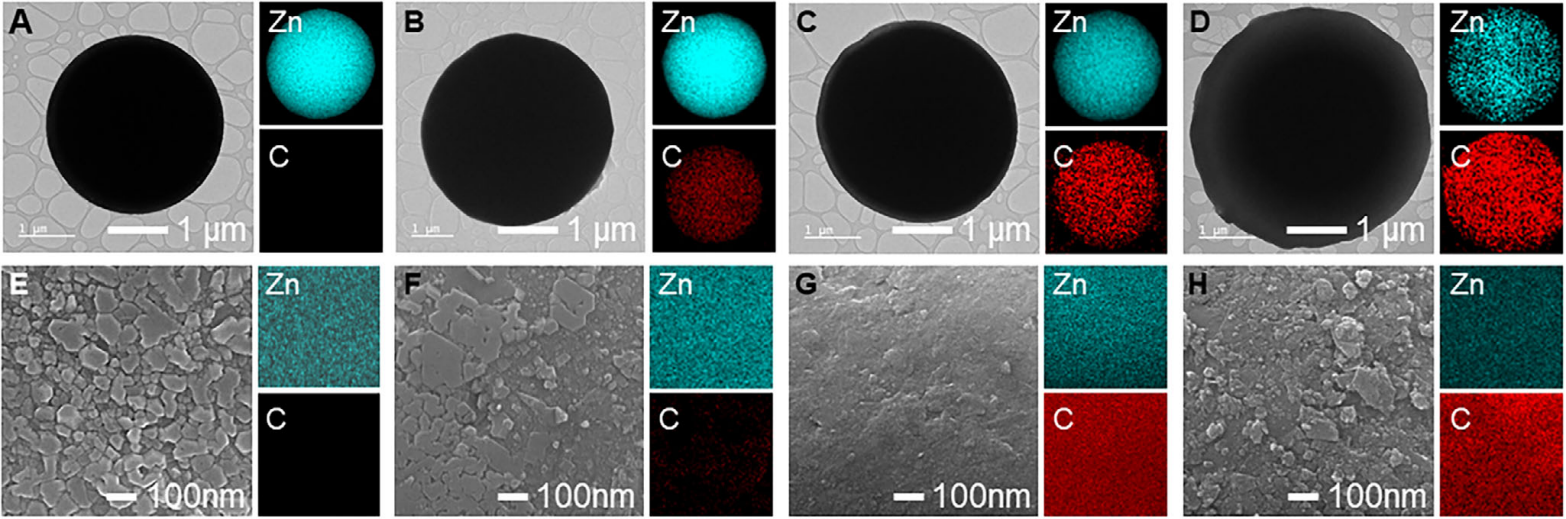
(A–D) TEM and EDX mapping images of (A) pristine Zn powder, (B) 1 wt.%, (C) 3 wt.%, and (D) 5 wt.% Gr_Zn samples. (E–H) SEM and EDX mapping images of (E) pristine Zn powder, (F) 1 wt.%, (G) 3 wt.%, and (H) 5 wt.% Gr_Zn samples.

Symmetric Zn metal coin cells were assembled to evaluate the electrochemical performance of the Gr_Zn. Figure 3A shows the Nyquist plots of the cells with Zn foil, P_Zn, and Gr_Zn. The result of Zn foil cell displayed a large semicircle, representing surface charge transfer resistance (R_ct_) around 4160 Ω. The powder electrode exhibited far reduced resistances, which are 116 Ω for Gr_Zn and 166 Ω for P_Zn, by a wide electrochemical surface area, indicating that ion transport is more favorable on the electrodes with Zn powders. The Zn metal nucleation barriers on the surface of powder Zn are also reduced, leading to the lower nucleation overpotential of 9.4 and 10.1 mV compared to 84.4 mV of the foil during initial zinc deposition at a current density of 1 mA cm^−2^ (Figure 3B). It is also noted that the graphene coating relieved a kinetic hindrance by reducing initial polarization and potential stabilizations between two powder‐type electrodes [49].

**FIGURE 3:**
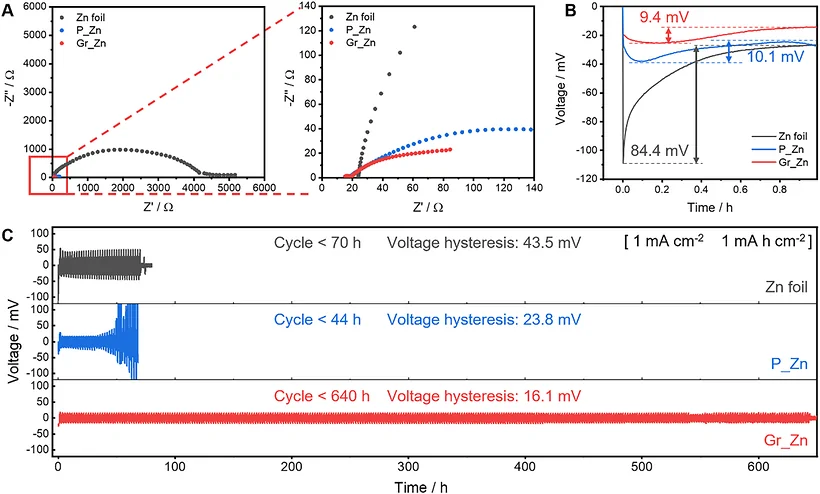
(A) Nyquist EIS plots of Zn||Zn, P_Zn||P_Zn, and Gr_Zn||Gr_Zn symmetric cells. (B and C) (B) Initial nucleation overpotential and (C) long‐term cycling voltage curves of Zn||Zn, P_Zn||P_Zn, and Gr_Zn||Gr_Zn symmetric cells at a current density of 1 mA cm^−2^ and an areal capacity of 1 mAh cm^−2^.

Figure 3C displays that Gr_Zn maintained stable cell operation over 640 h, compared to the foil and P_Zn. In detail, the Zn foil cell suffered internal short circuits due to dendrite growth before 70 h. And P_Zn exhibited a cell degradation caused by increased resistance from side reaction byproducts before 44 h under current density 1 mA cm^−2^ with a cycling capacity of 1 mAh cm^−2^. Further, as shown in Figure S6, Zn foil and P_Zn exhibited large average voltage hysteresis to 43.5 mV and 23.8 mV, respectively, even before degradation. Gr_Zn operates with the lowest voltage hysteresis of 16.1 mV for over 640 h. This is attributed to the ability of the graphene coating layer to suppress surface failures without resistive behaviors. To distinguish the effect from that of simple graphene blending, a graphene‐mixed Zn composite electrode was prepared. As seen in Figure S7, the graphene‐mixed Zn composite electrode does not exhibit these positive effects, indicating that the improvement originates from the coating rather than from simple graphene mixing. Figure S8 shows a similar consistency to Figure 3C, which is conducted under a current density of 2 mA cm^−2^ with a cycling capacity of 1 mAh cm^−2^. However, under the current density of 4 mA cm^−2^ with a cycling capacity of 2 mAh cm^−2^ in Figure S9, internal short‐circuits caused by dendrite growth occurred in P_Zn. This indicates that dendrites can also grow in Zn powder, suggesting that the graphene coating layer not only prevents side reactions but also suppresses dendrite growth.

It is also found that the graphene coating alters the solvation structure of the electrolyte contacting the surface. As illustrated in Figure 4A, typically, zinc ions in the electrolyte exist as hexahydrate [Zn(H_2_O)_6_]^2+^, which is called the solvent‐separated ion pair (SSIP) mode. When the electrolyte contacts to the graphene coating layer, free water molecules are displaced, leading to an increase in concentration within the coating layer, causing the SSIP mode to lose hydrated water and transition to the contact ion pair (CIP, [Zn^2+^(H_2_O)_5_∙OSO_3_2^−^]) mode [42, 48]. As shown in Figure 4B, the ν‐SO_4_^2−^ band (980–990 cm^−1^) of the graphene mixed electrolyte shifted to high frequency compared to the bare electrolyte, indicating a change from SSIP mode to CIP mode and suggesting that the zinc ions are interacting with fewer hydrated water in their solvation structure [50, 51, 52]. This alteration in solvation structure is possible since the graphene coating layer has hydrophobicity. As shown in Figure 4C–E, the contact angle of foil, P_Zn, and Gr_Zn are 109.9°, 84.2°, and 130.8°, respectively, indicating that P_Zn exhibits lower hydrophobicity than the foil due to its larger surface area. Gr_Zn, coated with graphene, which is well known for its hydrophobicity, exhibits significantly higher hydrophobicity than the others. This hydrophobic coating layer regulates the penetration of free water molecules into the layer, causing the electrolyte within the coating layer to alter from SSIP mode to CIP mode. Therefore, the solvation sheath becomes highly coordinated, reducing the reactive free water molecules generated during the desolvation process and suppressing side reactions such as HER and corrosion [53].

**FIGURE 4:**
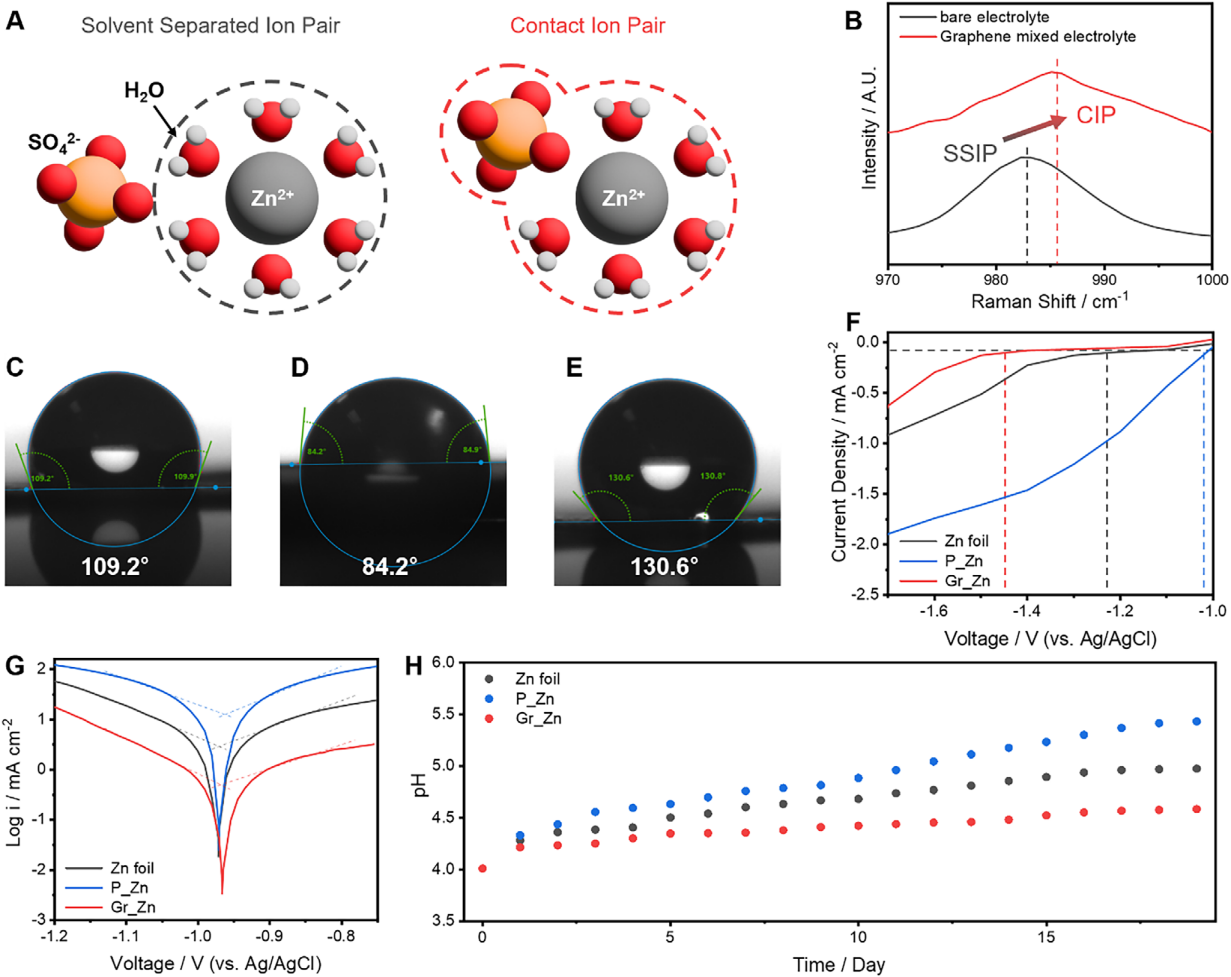
(A) Schematic illustrations of solvent‐separated ion pair (SSIP) mode (left) and contact ion pair mode (right). (B) Raman spectra of the bare electrolyte (bottom) and graphene mixed electrolyte (top). (C–E) Contact angle measurements of (C) Zn foil, (D) P_Zn, and (E) Gr_Zn. (F) Linear sweep voltammetry obtained in a 1 M Na_2_SO_4_ aqueous electrolyte. (G) Polarization curves of foil, P_Zn, and Gr_Zn with a 2 M ZnSO_4_ electrolyte. Dashed lines represent the Tafel fits. (H) pH changes of 2 M ZnSO_4_ electrolyte with soaking foil, P_Zn, and Gr_Zn.

A three‐electrode experiment was constructed to investigate the suppression of HER and corrosion in detail. To investigate the HER in different anodes, linear sweep voltammetry (LSV) was performed (Figure 4F). Gr_Zn exhibited the lowest onset voltage (−1.46 V) at 0.1 mA cm^−2^ compared to the Zn foil (−1.23 V) and P_Zn (−1.02 V). Additionally, Gr_Zn exhibited the lowest current density compared to foil and P_Zn, reflecting that Gr_Zn suppresses the HER. Polarization curves were measured to investigate interface corrosion (Figure 4G). Gr_Zn exhibited the highest corrosion potential (−0.966 V) and the lowest corrosion current density (i_corr_, 0.49 mA cm^−2^) compared to the Zn foil (−0.970 V, 2.85 mA cm^−2^) and P_Zn (−0.970 V, 12.4 mA cm^−2^), indicating less tendency of corrosion for Gr_Zn. Therefore, due to its hydrophobicity, the altered solvation structure within the graphene coating layer prevents HER, exhibiting anti‐corrosion properties. Side reactions suppression of the graphene coating layer also impacts the pH of the electrolyte. HER increases the pH of the electrolyte in conjunction with water electrolysis, as represented by reaction (Equation 1), while corrosion similarly elevates the pH of the electrolyte through reaction (Equation 2) [54, 55]. When the pH of the electrolyte increases, the concentration of OH^−^ ions increases, accelerating side reactions. Consequently, insulating byproducts from side reactions accumulate on the anode surface, leading to cell performance degradation. However, Gr_Zn suppresses the side reaction, exhibiting the lowest increase in electrolyte pH when immersed (Figure 4H).

(1) $$Zn+2H_{2}O\;\to \;Zn+2H^{+}+2OH^{-}\;\to \;Zn{\left(OH\right)}_{2}+H_{2}$$

(2) $$Zn\;-\;2e^{-}+4OH^{-}\;\to \;Zn{{\left(OH\right)}_{4}}^{2-}\;\to \;ZnO+H_{2}O+2OH^{-}$$

To investigate the crystal structure of Zn metal as cycling progresses, x‐ray diffraction (XRD) was employed. As seen in Figure 5A, the peak intensity ratio between (002) and (101) (I(002)/I(101)) of the foil decreases as cycling progresses, indicating that zinc deposition occurs preferentially on the (101) plane. P_Zn also shows a similar consistency to that of the foil (Figure 5B), which is attributed to the fact that the (101) plane has the strongest binding energy with Zn, leading to Zn deposition on the (101) plane, known as subsequent dendrite growth [56, 57, 58]. In contrast, as seen in Figure 5C, the I(002)/I(101) in Gr_Zn increases as cycling progresses, indicating that Zn deposition occurs preferentially on the (002) plane. Additionally, the foil and P_Zn exhibit peaks at 16.2°, 16.9°, 24.5°, 27.8°, and 32.8°, corresponding to zinc hydroxide sulfate (ZHS, Zn_4_(OH)_6_(SO_4_)·nH_2_O) as cycling progresses. In contrast, the absence of such a peak in Gr_Zn indicates that minimal side reaction by‐products that could hinder uniform Zn deposition is significantly suppressed.

**FIGURE 5:**
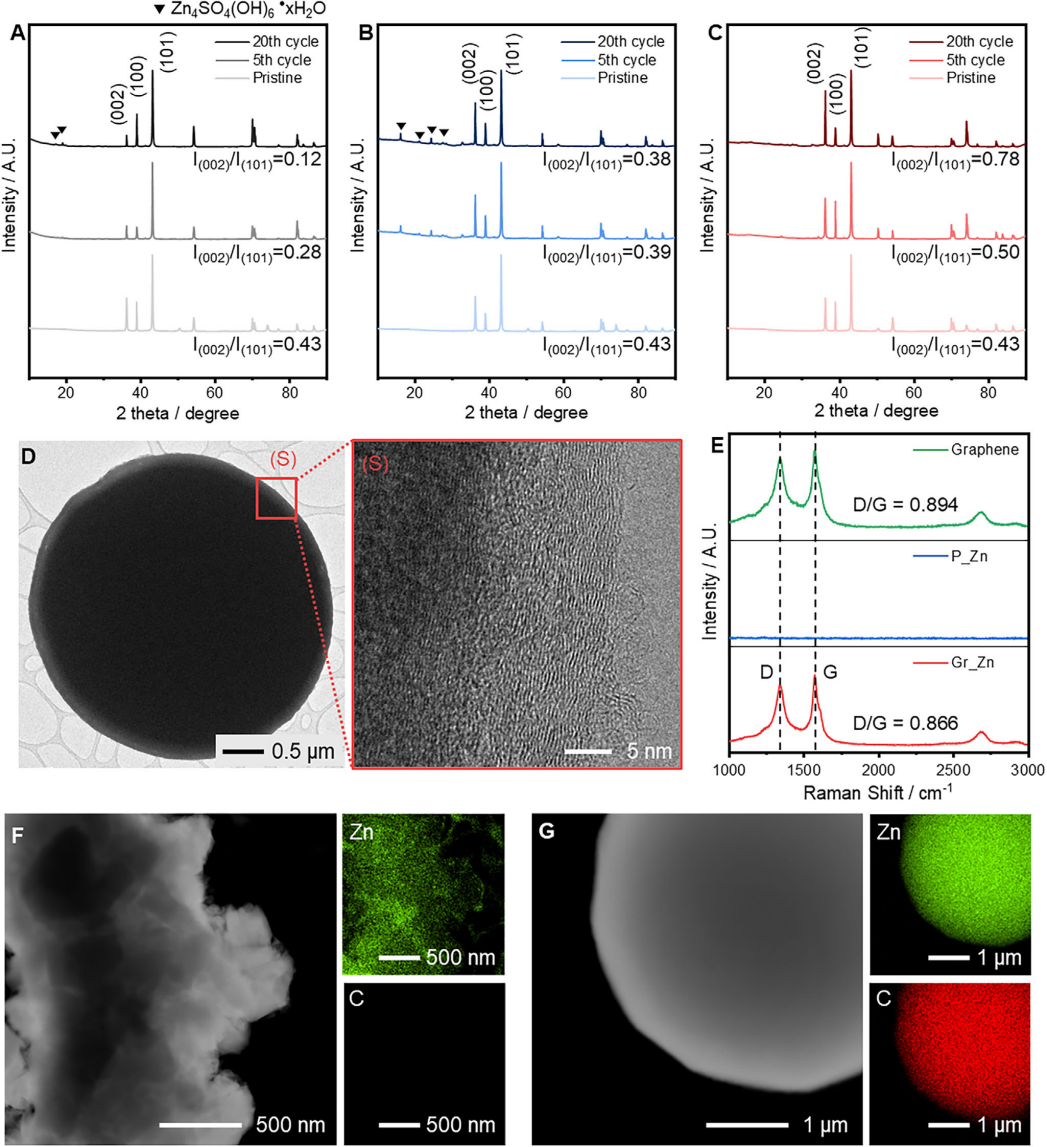
Ex situ XRD patterns of (A) foil, (B) P_Zn, and (C) Gr_Zn during cycling at a current density of 1 mA cm^−2^ with an areal capacity of 1 mAh cm^−2^. (D) TEM image of a Gr_Zn particle (left) and its high‐resolution TEM (HRTEM) image at the edge region (right). (E) Raman spectra of pristine graphene (top), P_Zn (middle), and Gr_Zn (bottom). After Zn deposition (1 mAh cm^−2^), TEM images and the corresponding EDS elemental mapping for Zn and C for (F) P_Zn and (G) Gr_Zn.

The preferential deposition of Zn onto the (002) plane is attributed to the minimal lattice mismatch between the graphene basal plane and the zinc (002) plane. As shown in Figure S10, the graphene basal plane exhibits a low lattice mismatch of only 5.6%, which facilitates hexagonal epitaxial growth originating from the (002) plane [59, 60, 61]. To verify that this preferential (002) deposition originates from the intimate graphene/Zn interface formed by the coating rather than from the mere presence of graphene, a control electrode was prepared by simply mixing the same amount of graphene (3 wt.%) with Zn powder without the mechanofusion coating. The mixed electrode showed no sustained increase in I(002)/I(101) upon cycling (Figure S11), and ZHS peaks emerged after cycling, as observed for P_Zn, confirming that the epitaxial growth of Zn requires the graphene coating layer on the particle surface. The high‐resolution TEM images in Figure 5D show graphene layers on the surface of Gr_Zn, indicating that the basal plane of graphene is exposed outward after coating. Additionally, the Raman spectroscopy spectra in Figure 5E reveal that Gr_Zn has a similar D/G ratio, representing the ratio of the D‐band (1350 cm^−1^) to the G‐band (1580 cm^−1^), with pristine graphene, indicating that defects are not formed in the graphene structure after mechanofusion coating process [62, 63, 64]. Therefore, after coating, graphene exposes its basal plane with no defects formation and a low lattice mismatch with the (002) plane, enabling zinc deposition along the (002) plane on Gr_Zn.

The (002) plane, exhibiting the lowest surface energy, allows for uniform nucleation, enabling horizontal and flat Zn deposition. TEM image of P_Zn represents that Zn was deposited non‐uniformly after 1 mAh cm^−2^ zinc deposition (Figure 5F). In contrast, TEM and EDS mapping images of Gr_Zn show that zinc is deposited beneath the graphene and flatly under the same conditions (Figure 5G). Further, Figure S12 shows images of Gr_Zn obtained from ex‐situ TEM during Zn deposition after Zn stripping, which is identical to those of full‐cell operation. When Zn is stripped from Gr_Zn, the graphene coating layer maintains its spherical shape while the Zn metal inside is removed, creating empty space. When Zn is deposited after stripping, it is deposited onto the residual Zn remaining within the graphene coating layer, filling the empty spaces. Subsequently, additional Zn deposition results in flat deposition. As a result, the flat zinc deposition suppresses dendrite growth, improving the electrochemical performance of Gr_Zn.

SEM characterized the surface morphology changes during cycling. As seen in Figure 6A, the initially smooth surface of the foil exhibited dendrite formation after just 5 cycles, along with the appearance of ZHS on the surface. After 20 cycles, further dendrite growth was observed, with a significant increase in dendrites. Similarly, in P_Zn, dendrite formation was observed after just 5 cycles, with the surface predominantly covered by ZHS compared to its initial morphology. After 20 cycles, a significant presence of dendrites was observed extending beyond the ZHS, indicating dendrite growth. In contrast, Gr_Zn exhibited no morphology changes during the first 5 cycles, and neither dendrites nor ZHS were observed even after 20 cycles, indicating that stable cycling was maintained. Figure 6B shows the analysis of the chemical composition of the zinc anode surface after 20 cycles under the same conditions, using deconvoluted XPS spectra. The peaks at 1022.3, 1022.7, and 1023.5 eV in the Zn 2p_3/2_ spectra correspond to the bonding states of side reaction byproducts ZnO, Zn(OH)_2_, and ZnSO_4_, respectively, while the peak at 1021.6 eV corresponds to fresh zinc [65]. Analyses of the fresh zinc remaining on the zinc anode surface show that only 9.4% and 5% remain on the foil and P_Zn, respectively, after 20 cycles. In contrast, 80% of fresh zinc remains on the surface of Gr_Zn after 20 cycles, significantly more than on the foil and P_Zn. This is supported by O 1s spectra, where the side reaction byproducts ZnO, Zn(OH)_2_, and ZnSO_4_ (532.0, 532.2, 533.0 eV, respectively) appear in the same proportions as in the Zn 2p_3/2_ spectra [65]. In contrast to the foil and P_Zn, which only show peaks corresponding to side reaction byproducts, Gr_Zn shows almost no such side reaction byproducts, with 84 at.% of the surface covered by carboxyl functional groups from the graphene coating layer.

**FIGURE 6:**
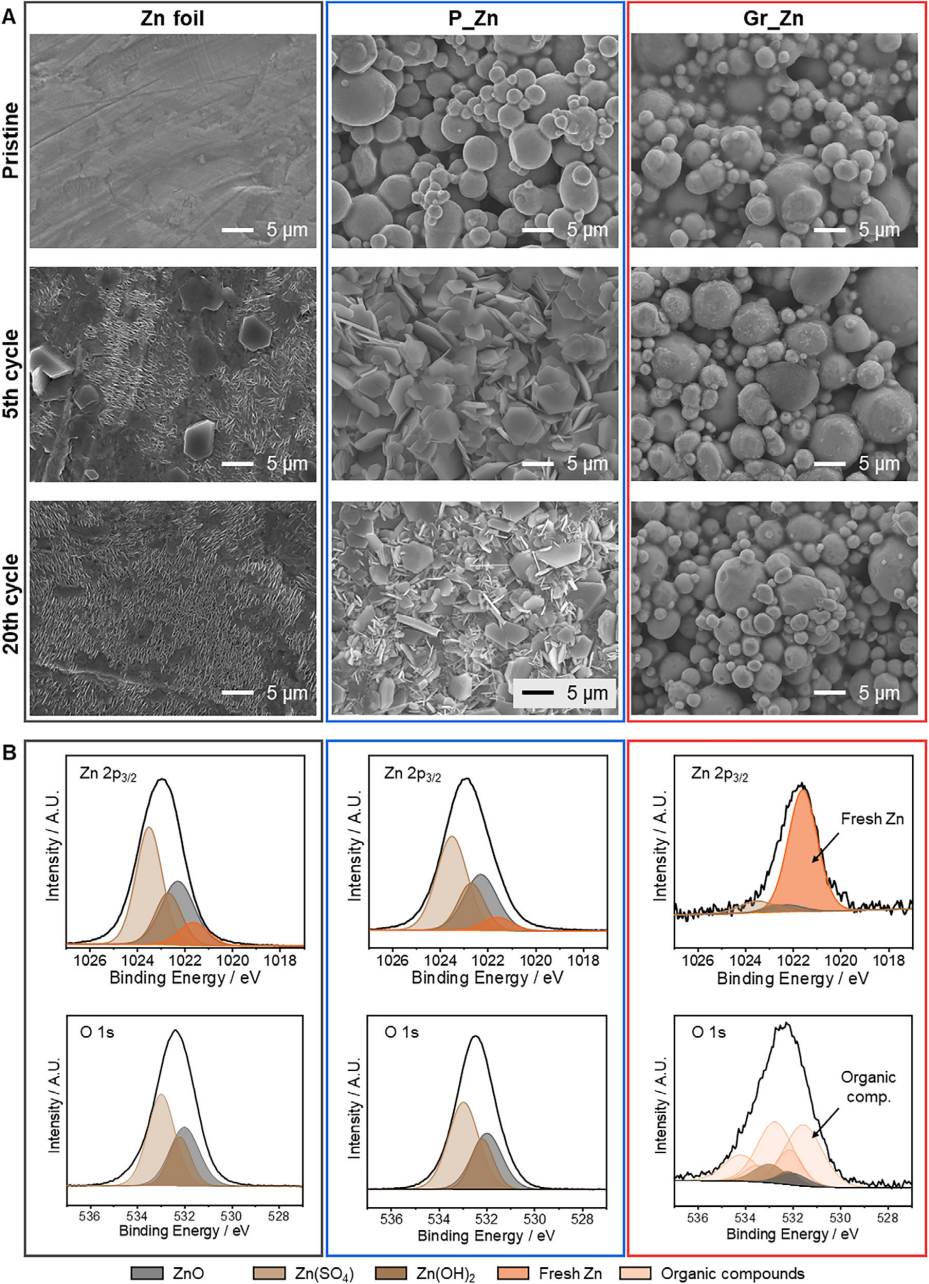
(A) Ex situ SEM images of foil (top), P_Zn (middle), and Gr_Zn (bottom) during cycling at a current density of 1 mA cm^−2^ with an areal capacity of 1 mAh cm^−2^. (B) Zn 2p_3/2_ (left) and O 1s (right) XPS spectra of foil, P_Zn, and Gr_Zn after 20 cycles at a current density of 1 mA cm^−2^ with an areal capacity of 1 mAh cm^−2^.

Due to the graphene coating layer suppressing side reactions and dendrite growth, the Gr_Zn can operate stably under various conditions. Figure 7A shows the rate capability of the symmetric cell measured at current densities of 1, 2, 4, 8, and 10 mA cm^−2^ with a cycling capacity of 1 mAh cm^−2^. Compared to the foil, in which short circuits occur at a current density of 2 mA cm^−2^, the composite electrode exhibits superior rate capability with its larger surface area facilitating ion transport. However, P_Zn suffered cell degradation due to internal resistance at a current density of 10 mA cm^−2^, caused by side reaction byproducts and dead zinc. Gr_Zn, which suppressed side reactions and dendrite growth, achieved stable operation even at a high current density of 10 mA cm^−2^, exhibiting the lowest voltage hysteresis across all current densities, as seen in Figure 7B and Figure S13. Furthermore, when returning to a lower current density, the cell continued to operate stably without cell degradation. The Nyquist EIS in Figure 7C and S14 supports these results. Since Gr_Zn has a large surface area and inhibits side reactions and dendrite growth, Gr_Zn has the lowest R_ct_ after the first cycle and continues to show the same consistency in subsequent cycles, demonstrating that ion transport is most favorable in Gr_Zn during cycling. At low temperatures, the kinetics of zinc ions decrease, making uniform zinc deposition difficult and accelerating dendrite growth. However, due to the graphene coating layer suppressing dendrite growth, Gr_Zn could operate stably even at low temperatures of −10°C under a current density of 1 mA cm^−2^ with a cycling capacity of 1 mAh cm^−2^ (Figure S15).

**FIGURE 7:**
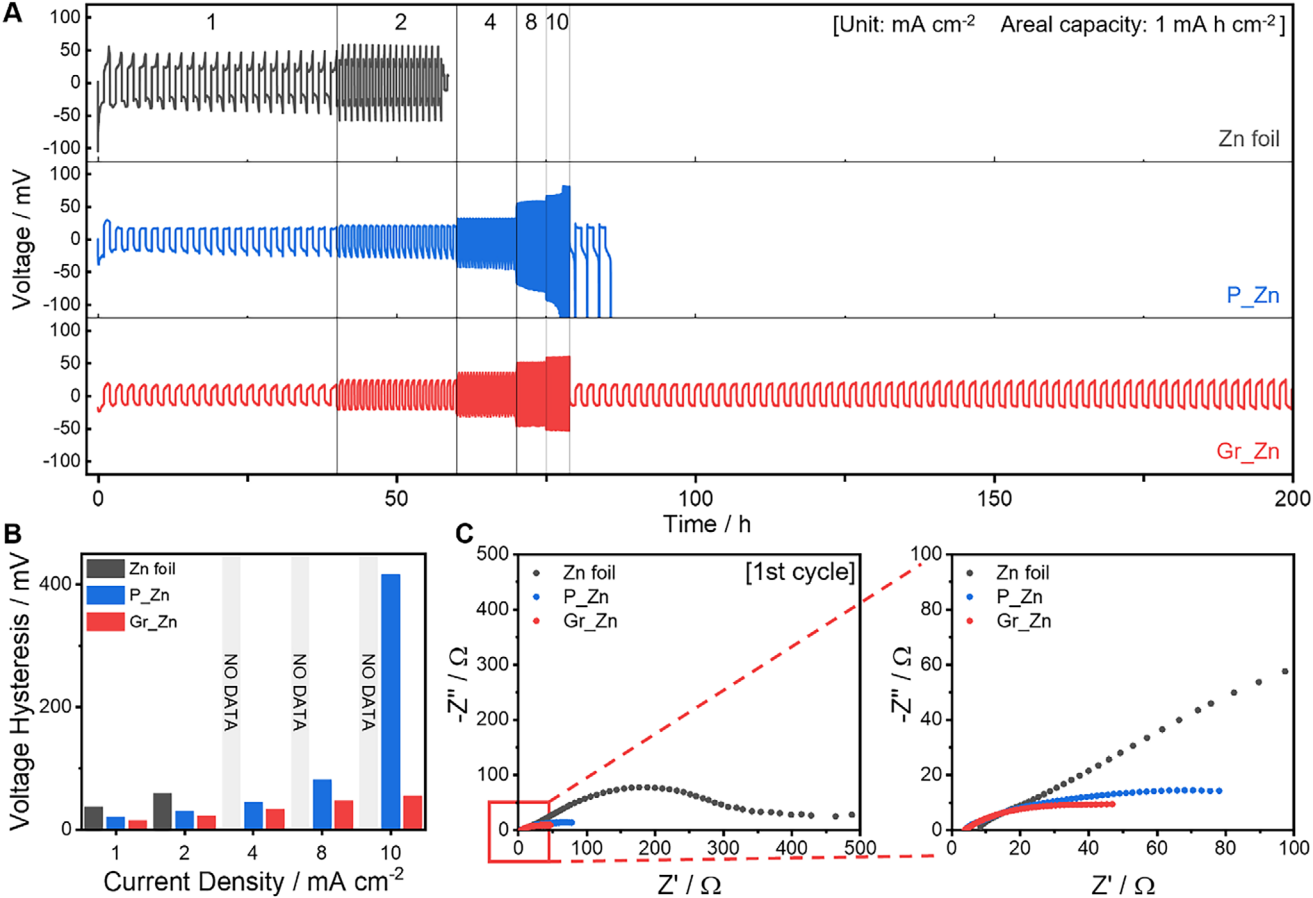
(A) Rate capability of Zn||Zn (top), P_Zn||P_Zn (middle), and Gr_Zn||Gr_Zn (bottom) symmetric cells at current densities of 1, 2, 4, 8, and 10 mA cm^−2^ with a cycling capacity of 1 mAh cm^−2^, and (B) its voltage hysteresis at each current density. (C) Nyquist EIS plots of Zn||Zn, P_Zn||P_Zn, and Gr_Zn||Gr_Zn symmetric cells.

In this way, to verify whether Gr_Zn can be performed in a full cell, electrochemical tests were conducted using NH_4_V_4_O_10_ (NVO) as the cathode. Figure 8A shows the initial cyclic voltammetry (CV) measured at a scan rate of 50 µV sec^−1^ in the voltage range of 0.4–1.4 V. The composite electrodes with a large surface area show smaller polarization at all redox peaks in the CV compared to the foil. Notably, Gr_Zn exhibits lower polarization than P_Zn, as the suppression of side reactions prevents by‐product formation during the 12‐h resting period. Furthermore, Gr_Zn shows the highest current density at all redox peaks, indicating that zinc ion migration is more facilitated on Gr_Zn surface during charge and discharge. Consequently, Gr_Zn delivers the smallest polarization and the highest specific capacity of 247.2 mAh g^−1^ (foil: 217.1 mAh g^−1^, P_Zn: 243.6 mAh g^−1^), as demonstrated in the initial galvanostatic charge/discharge profiles at 2 A g^−1^ (Figure S16). Figure 8B presents the discharge‐specific capacity as a function of cycle number at a current density of 2 A g^−1^. Gr_Zn achieved the highest specific capacity and capacity retention of 68.1% at the 100th cycle, in contrast to the foil, which showed a capacity retention of 20.5%, and P_Zn, which showed a capacity retention of 12.4%, indicating that Gr_Zn operates stably in a full cell. As a result, Gr_Zn exhibits the highest initial Coulombic efficiency (C.E.) of 81.2% compared to the foil (76.0%) and P_Zn (81.1%), and it maintains a C.E. of 99.6% as the cycles progress (foil: 98.9%, P_Zn: 98.7%) (Figure 8C). Moreover, Gr_Zn maintains the highest specific capacity across all current densities of 0.5, 1, 2, 4, 10, and 20 A g^−1^, demonstrating excellent rate capability in a full cell (Figure 8D and Figure S17).

**FIGURE 8:**
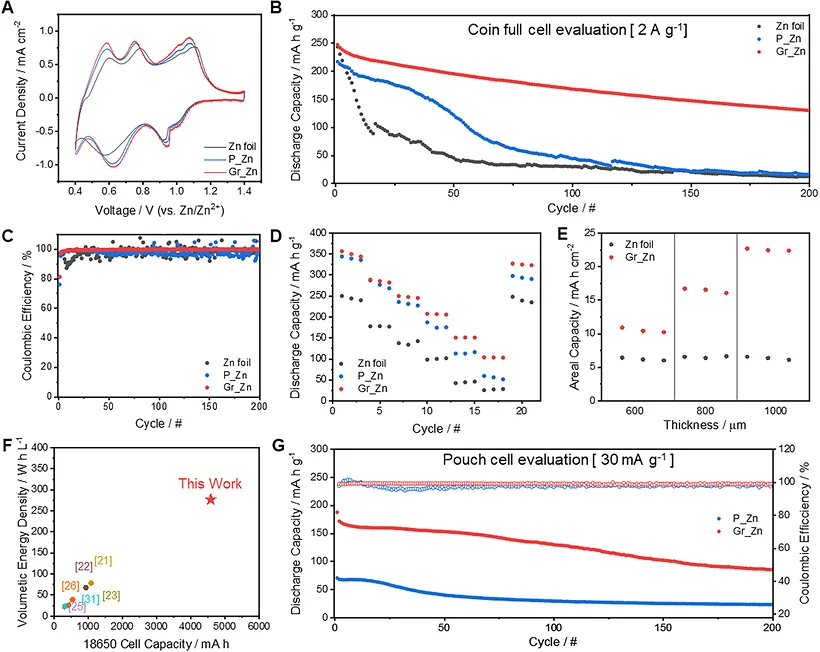
(A) CV curves of Zn||NVO, P_Zn||NVO, and Gr_Zn||NVO at a scan rate of 50 µV s^−1^ within the voltage range of 0.4–1.4 V. (B) Cycling performance of coin full cells using Zn||NVO, P_Zn||NVO, and Gr_Zn||NVO at 2 A g^−1^ (NVO areal capacity: 1.2 mAh cm^−2^) and their (C) Coulombic efficiencies and (D) rate capabilities. (E) Areal capacity according to cathode thickness at a current density of 0.15 A g^−1^. (F) Summary of the volumetric capacity calculated from the actual cell configuration and the corresponding projected capacity when scaled to an 18650‐cell configuration. (G) Cycling performance of Gr_Zn||MnO_2_ and P_Zn||MnO_2_ pouch cells (MnO_2_ areal capacity: 3.1 mAh cm^−2^).

The 2D interface of the foil with the electrolyte limits the pathways for ion transport. Also, side reaction byproducts and dead zinc during the stripping/deposition processes hinder zinc migration. These limit the migration of a large amount of zinc ions simultaneously in the foil [66, 67]. Consequently, foil is hard to implement in thick electrode batteries, presenting a substantial obstacle to high‐energy density batteries. However, Gr_Zn enables substantial zinc ion transport by forming a 3D interface with the electrolyte, suppressing dendrite growth and side reactions. Therefore, Gr_Zn can be applied in thick electrode batteries for high‐energy density batteries. To verify this, we operated in‐house‐designed full cells (Figure S18) employing thick NVO cathodes with thicknesses of 600, 800, and 1000 µm, corresponding to loading levels of 17, 23, and 32 mAh cm^−^^2^, respectively, while maintaining the same electrode density. As shown in Figure 8E, the foil does not exceed an areal capacity of 7.5 mAh cm^−^^2^, exhibiting similar areal capacity across all thicknesses of cathodes. In contrast, Gr_Zn exhibits significantly higher areal capacities of 10.9, 16.7, and 22.7 mAh cm^−^^2^ when applied to cathodes of 600, 800, and 1000 µm thicknesses, respectively (Figure S19). As a result, this demonstrates the substantial migration of zinc ions in Gr_Zn, indicating its applicability to thick electrode batteries (Figure S20). Assuming the application of the thick electrode in the 18650 battery, capacity and volumetric energy density at the 18650 battery were calculated using Equations (3)–(5) to compare with other zinc composite electrode research.

(3) $$\begin{array}{ccc} & & \mathrm{mass}\;\mathrm{of}\;\mathrm{cath}\mathrm{ode}=\mathrm{volu}\mathrm{me}\;\mathrm{of}\;18650\\ & & \;\;\;\;\;\;\times \;\frac{\mathrm{thic}\mathrm{kness}\;\mathrm{of}\;\mathrm{cath}\mathrm{ode}\;\mathrm{elec}\mathrm{trode}}{\mathrm{total}\;\mathrm{thic}\mathrm{kness}\;\mathrm{of}\;\mathrm{comp}\mathrm{onen}\mathrm{ts}}\\ & & \;\;\;\;\;\;\times \;\mathrm{cath}\mathrm{ode}\;\mathrm{dens}\mathrm{ity}\\ & & \mathrm{comp}\mathrm{onen}\mathrm{ts}:\mathrm{cathode}\;\mathrm{and}\;\mathrm{anode}\;\mathrm{elec}\mathrm{trode},\\ & & \;\;\mathrm{cathode}\;\mathrm{and}\;\mathrm{anode}\;\mathrm{curr}\mathrm{ent}\;\mathrm{coll}\mathrm{ector},\;\mathrm{sepa}\mathrm{rator} \end{array}$$

(4) capacity=massofcathode×measuredspecificcapacity

(5) $$volumetric\;energy\;density=\frac{capacity\;\times \;nominal\;voltage}{volume\;of\;18650}$$

Assuming a separator thickness of 20 µm, the calculations indicate that Gr_Zn exhibits a much higher capacity and volumetric energy density than other composite electrode research (Figure 8F). Additionally, to evaluate the potential for commercialization, single‐layer Zn||MnO_2_ pouch cells were fabricated (energy and power densities of ∼91 Wh kg^−^^1^ and ∼15 W kg^−^^1^, respectively, based on the total electrode composite mass). As shown in Figure 8G, Gr_Zn achieved a higher initial capacity (187.8 mAh g^−1^) and initial Coulombic efficiency (99.0%) compared to P_Zn at a current density of 30 mA g^−1^. At 100 cycles, Gr_Zn exhibited a high cycle retention of 75.6%, along with an impressive average Coulombic efficiency of 99.2% during this period. Furthermore, as shown in Figure S21, the pouch cell using P_Zn exhibited swelling due to hydrogen gas evolution caused by side reactions during the charge/discharge cycles. In contrast, Gr_Zn demonstrated stable operation without any swelling of the pouch cell. Therefore, Gr_Zn, which operates stably in both thick electrode cells and pouch cells, can potentially contribute to the commercialization of AZMBs.

## 3 Conclusions

In this study, we successfully fabricated a Gr_Zn anode via a mechanofusion‐based dry‐coating technique, effectively addressing the inherent limitations of aqueous zinc‐metal batteries (AZMBs). The hydrophobic graphene layer significantly suppresses parasitic side reactions by modulating the electrolyte's solvation structure. Compared to bare Zn foil and P_Zn, Gr_Zn exhibits superior electrochemical stability, sustaining over 640 h of cycling with minimal voltage hysteresis and exceptional rate capability. Furthermore, the low lattice mismatch between the graphene basal plane and the Zn (002) plane facilitates uniform, dendrite‐free zinc deposition through epitaxial growth. These results demonstrate that the Gr_Zn anode design overcomes critical hurdles in AZMBs, providing high energy density and long‐term durability. Moreover, its mechanical robustness offers the versatility required for practical applications, such as thick‐electrode systems and flexible and foldable electronic devices. This work provides a robust strategy for the commercialization of AZMBs, effectively bridging the gap between high‐performance interface engineering and industrial scalability.

## 4 Experimental Section

### 4.1 Preparation of Gr_Zn

Gr_Zn was prepared using the mechanofusion dry coating method. 1, 3, 5 wt.% graphene (Sigma‐Aldrich) and P_Zn (Sigma‐Aldrich) were mixed and coated with a dry coater under 3000 rpm for 10 min. Obtained Gr_Zn was mixed with a Polyvinylidene fluoride (PVdF) binder at a weight ratio of 95: 5. 1‐Methyl‐2‐pyrrolidone (NMP, Sigma‐Aldrich) was added and mixed homogeneously using a C‐mixer to produce a slurry. The homogeneous slurry was cast on Copper foil. Then, it was dried at 120°C for 10 h under vacuum and compressed using a roll press.

### 4.2 Preparation of Cathode

NVO was synthesized using the hydrothermal method. The 1.170 g of NH_4_VO_3_ powder (Sigma Aldrich) was dispersed into 50 mL of deionized water under 80°C for 15 min. Then, 1.891 g of Oxalic acid (Sigma‐Aldrich) was added, and the solution was stirred for 40 min until the color changed to black–green. After stirring, the solution was poured into an autoclave and heated at 140°C for 48 h. After naturally cooling to room temperature, the powder was collected using filter paper and washed with deionized water. The final NVO powder was obtained by drying at 80°C for 12 h under vacuum. Obtained NVO was mixed with Super‐P and a PVdF binder at a weight ratio of 80: 10: 10. NMP was added and mixed homogeneously using a C‐mixer to produce a slurry. The homogeneous slurry was cast on carbon paper. Then, it was dried at 120°C for 10 h under vacuum and compressed using a roll press. The thick electrode was fabricated by mixing NVO with Super‐P and a Polytetrafluoroethylene (PTFE, Sigma–Aldrich) binder at a weight ratio of 80: 10: 10. Deionized water was added and mixed to produce a homogeneous mixture. The homogeneous mixture was compressed using a hand press and then dried at 120°C for 10 h under vacuum. Then, the thick electrode was obtained by compressing with a roll press with thicknesses of 600, 800, and 1000 µm.

### 4.3 Material Characterizations

TEM and EDS mapping images were obtained on a high‐performance EDS embed transmission electron microscope (JEOL, JEM‐F299). XPS spectra were recorded using an x‐ray photoelectron spectrometer (Thermo‐Scientific, ESCALAB 250Xi), and all binding energies were calibrated based on the C 1s peak (285 eV). SEM and EDS mapping images were obtained on a field emission scanning electron microscope (JEOL, JSM‐IT800). Raman spectra were recorded using a Raman spectrometer (Horiba, LabRam ARAMIS) with a 532 nm laser. The contact angles of the zinc anodes were measured using a drop shape analyzer (Kruss, DSA25). The pH levels of the 2 M ZnSO_4_ aqueous electrolytes immersed with zinc anodes were measured using an eco titrator (Metrohm). XRD patterns were recorded using a high‐power x‐ray diffractometer system (Bruker Corporation, D8 ADVANCE).

### 4.4 Electrochemical Measurements

LSV and polarization curves were performed on a battery cycler (WonaTech, WBCS‐300, South Korea) using a three‐electrode cell configuration, with the zinc anodes (0.2 × 0.2 cm) as the working electrode, Pt mesh as the counter electrode, and Ag/AgCl (in 3.5 M KCl) as the reference electrode. LSV was measured in a 1 M Na_2_SO_4_ aqueous electrolyte from −1.0 to −1.8 V at a scan rate of 10 mV sec^−1^. The polarization curve was measured in a 2 M ZnSO_4_ aqueous electrolyte from −0.5 to −1.5 V at a scan rate of 1 mV sec^−1^. The electrochemical performance of half cell and full cell was evaluated using 2032 coin‐type cells, with 2 M ZnSO_4_ aqueous electrolyte and glass fiber as a separator. EIS was measured on a Zive Lab (WonaTech, MP2, South Korea) with a frequency range from 100 kHz to 0.01 Hz and a voltage amplitude of 5 mV at open‐circuit voltage. Cyclic galvanostatic tests and CV were performed on a battery cycler (WonaTech, WBCS‐300, South Korea). The areal capacity of the Gr_Zn anode was 7 mAh cm^−2^ for the standard full cells (cathode: 1.2 mAh cm^−2^; N/*p* = 5.8) and 34 mAh cm^−2^ for the thick‐electrode cells (cathode: 17, 23, and 32 mAh cm^−2^; N/*p* = 2.0, 1.5, and 1.06, respectively).

## Author Contributions

**Daebeom Jeong**: investigation, Writing – review and editing, visualization, validation, methodology, data curation, formal analysis. **Hun‐Gi Jung**: project administration, supervision, investigation. **Jongsoon Kim**: investigation, supervision, project administration. **Goojin Jeong**: methodology, supervision, investigation, formal analysis, resources, writing – review and editing. **Junyoung Mun**: conceptualization, writing – original draft, writing – review and editing, methodology, supervision, resources, funding acquisition. **Kijung Kim**: data curation, investigation. **Van‐Chuong Ho**: investigation. **Dongju Kim**: methodology, software, data curation, investigation, formal analysis, visualization, validation, writing – original draft. **Jung Ho Kim**: investigation, writing – review and editing, visualization, conceptualization, supervision, resources.

## Conflicts of Interest

The authors declare no conflicts of interest.

## Supporting information




**Supporting File**: advs77287‐sup‐0001‐SuppMat.docx.

## Data Availability

The data that support the findings of this study are available from the corresponding author upon reasonable request.
