# Supplementary material for: Basal Plane Graphene Coated Zn Powder for High Performance Aqueous Zn Metal Batteries
Source: Adv Sci (Weinh). 2026 Aug 20;13(62):e77287. doi: 10.1002/advs.77287 (PMC13491366; doi:10.1002/advs.77287)
Supplement: Supplementary file 1 [file ADVS-13-e77287-s001.docx]

Supporting Information

Basal plane Graphene coated Zn powder for high performance aqueous Zn metal batteries

Dongju Kim, Daebeom Jeong, Kijung Kim, Van-Chuong Ho, Jongsoon Kim, Hun-Gi Jung, Jung Ho Kim*, Goojin Jeong*, Junyoung Mun*

Figure S1. Digital photographs of (A) bent state, (B) one-folded state, (C) two-folded, and (D) unfolded after folding

Figure S2. TEM and EDS mapping images of (A) P_Zn, (B) 1 wt.% Gr_Zn, (C) 3 wt.% Gr_Zn, and (D) 5 wt.% Gr_Zn samples.

Figure S3. Accumulated EDS mapping images obtained from TEM-EDS on the particle surfaces of (A) 1 wt.% Gr_Zn, (B) 3 wt.% Gr_Zn, and (C) 5 wt.% Gr_Zn samples. (Zn: cyan, and C: red)

Figure S4. XPS Spectra of C 1s, O 1s, and Zn 2p3/2 for P_Zn, 1 wt.% Gr_Zn, 3 wt.% Gr_Zn, and 5 wt.% Gr_Zn samples.

Figure S5. SEM images of (A) P_Zn, (B) 1, (C) 3, and (D) 5 wt.% Gr_Zn

Figure S6. Voltage profiles of Zn||Zn, P_Zn||P_Zn, and Gr_Zn||Gr_Zn symmetric cells at a current density of 1 mA cm^-2^ with a cycling capacity of 1 mAh cm^-2^ at (A) initial 5 cycles and (B) from the 250th to 255th cycle.

Figure S7. Long-term cycling of Gr mixed Zn||Gr mixed Zn and Gr_Zn||Gr_Zn symmetric cells at a current density of 1 mA cm^-2^ with a cycling capacity of 1 mAh cm^-2^.

Figure S8. Long-term cycling of Zn||Zn, P_Zn||P_Zn, and Gr_Zn||Gr_Zn symmetric cells at a current density of 2 mA cm^-2^ with a cycling capacity of 1 mAh cm^-2^.

Figure S9. Long-term cycling of Zn||Zn, P_Zn||P_Zn, and Gr_Zn||Gr_Zn symmetric cells at a current density of 4 mA cm^-2^ with a cycling capacity of 2 mAh cm^-2^.

Figure S10. Crystal structural illustration of Zn (002) and graphene basal plane.

**Figure S11.** Ex-situ XRD patterns of the graphene-mixed Zn electrode (3 wt.%, simply mixed without mechanofusion coating) before cycling and after 5 and 20 cycles at a current density of 1 mA cm-2 with an areal capacity of 1 mAh cm-2. ZHS reflections emerge after cycling.

Figure S12. TEM images of 2 mAh cm^-2^ Zn plated Gr_Zn (left), 2 mAh cm^-2^ Zn deposition after 2 mAh cm^-2^ Zn plated (middle), and 3 mAh cm^-2^ Zn deposition after 2 mAh cm^-2^ Zn plated (right).

Figure S13. Voltage profiles of Zn||Zn, P_Zn||P_Zn, and Gr_Zn||Gr_Zn symmetric cells at current densities of (A) 1 mA cm^-2^, (B) 2 mA cm^-2^, (C) 4 mA cm^-2^, (D) 8 mA cm^-2^, (E) 10 mA cm^-2^ with a cycling capacity of 1 mAh cm^-2^.

Figure S14. Nyquist EIS plot of Zn||Zn, P_Zn||P_Zn, and Gr_Zn||Gr_Zn symmetric cells after (A) 5 cycles and (B) 20 cycles at a current density of 1 mA cm^-2^ with a cycling capacity of 1 mAh cm^-2^.

Figure S15. Long-term cycling of Zn||Zn, P_Zn||P_Zn, and Gr_Zn||Gr_Zn symmetric cells at a current density of 2 mA cm^-2^ with an areal capacity of 1 mAh cm^-2^ at a low temperature of -10 °C.

Figure S16. (A) initial cycle and (B) 50th cycle galvanostatic charge and discharge curves of Zn||NVO, P_Zn||NVO, and Gr_Zn||NVO full cells at a current density of 2 A g^-1^.

Figure S17. Galvanostatic charge and discharge curves of (A) Zn||NVO, (B) P_Zn||NVO, and (C) Gr_Zn||NVO full cells at a current density of 0.5, 1, 2, 4, 10, and 20 A g^-1^.

Figure S18. (A) Photographs of a homemade cell used for thick-electrode evaluation and (B) its cross-sectional schematic illustration.

Figure S19. Digital photos of NVO thick electrodes with thicknesses of (A) 600 μm, (B) 800 μm, and (C) 1000 μm.

Figure S20. Galvanostatic charge and discharge curves of Zn||NVO and Gr_Zn||NVO full cells with (A) 600 μm, (B) 800 μm, and (C) 1000 μm NVO cathodes at a current density of 0.15 A g^-1^.

Figure S21. Photographs of the pouch cells using (A) P_Zn and (B) Gr_Zn as anode after 100 cycles.
